# Comparison of different models to calculate the viscosity of biogas and biomethane in order to accurately measure flow rates for conformity assessment

**DOI:** 10.1038/s41598-021-81052-7

**Published:** 2021-01-18

**Authors:** Karine Arrhenius, Oliver Büker

**Affiliations:** Research Institutes of Sweden AB (RISE), Brinellgatan 4, 50462 Borås, Sweden

**Keywords:** Chemistry, Energy science and technology, Renewable energy

## Abstract

The study presents an optimised method to correct flow rates measured with a LFE flowmeter pre-set on methane while used for gas mixtures of unknown composition at the time of the measurement. The method requires the correction of the flow rate using a factor based on the viscosity of the gas mixtures once the composition is accurately known. The method has several different possible applications inclusive for the sampling of biogas and biomethane onto sorbent tubes for conformity assessment for the determination of siloxanes, terpenes and VOC in general. Five models for the calculation of the viscosity of the gas mixtures were compared and the models were used for ten binary mixtures and four multi-component mixtures. The results of the evaluation of the different models showed that the correction method using the viscosity of the mixtures calculated with the model of Reichenberg and Carr showed the smallest biases for binary mixtures. For multi-component mixtures, the best results were obtained when using the models of Lucas and Carr.

## Introduction

Non-conventional gases (biogas, biomethane, hydrogen, syngas and mixtures with natural gas) may contain impurities that can damage existing gas infrastructures and/or vehicles if they are present in too high levels in the gases. Example for these impurities for biomethane are siloxanes and sulphur compounds. Through the combustion process in engine, siloxanes can be transformed into silicon dioxide. Consequently, a silica layer can be formed and deposited on the spark plug, cylinder, and impeller, causing the wear and damage of the engine parts and shorten the life of the engine^1^. Through the combustion process in engine, sulfur compounds form sulfur dioxide which has an inhibitory and aging impact on catalysts^2^.

Specifications developed for biomethane; ISO16723-1^3^ and ISO16723-2^4^ set maximum authorized concentrations for some of these impurities. Consequently, robust and reliable methods are required to enable conformity assessment. Analytical methods have been developed for many of these impurities^5,6^. A thermal desorption–Gas chromatography–mass spectrometry method (TD–GC–MS, EN ISO 16017-1^7^) is the analytical method proposed for the quantification of siloxanes in biomethane in ISO16723-2. According to this method, the first step is to collect the gas onto adequate sorbents on which the siloxanes adsorb while the matrix passes through unretained. The sorbents are then subsequently thermally desorbed; step which allows the impurities to be released from the sorbents. If possible, onsite, the gas is collected directly onto the sorbents to avoid risk of losing the impurities by adsorption onto the walls of intermediate vessels such as a sampling bags or cylinders.

This operation requires a known volume of gas to be collected onto the sorbent tube at an adequate flow rate preferably using a flowmeter. The optimum flow rate used to collect VOCs (inclusive siloxanes and sulfur compounds) on ¼-inch (6.35 mm) O.D. tubes have been reported to be 50 mL/min for air, no value has yet been published for biogas or biomethane and flow rates from 10 to 200 mL/min are typically applied. Higher sampling flow rates in excess of 200 mL/min can also be used for short term monitoring (e.g., 10 min)^8^.

However, biogas and biomethane composition varies with respect to the main components depending on the process. Methane content varies between 40 and 99 vol% by volume and carbon dioxide content varies from less than 1 vol-% to 60 vol-%. Other compounds such as nitrogen, water vapour and hydrogen sulphide can be present in the gas at levels between some ppm and up to some percent. Finally, even hydrocarbons such as ethane and propane can be present in the gas for instance if some natural gas has been mixed with biomethane. Generally, the exact composition is not known until a complete analysis is performed in a laboratory.

An example of flowmeters that can be used for the purpose of sampling biogas and biomethane onto sorbent tubes is a Laminar Flow Element (LFE). The volumetric flow rate is determined by creating a pressure drop across an unique restriction, known as LFE, and measuring differential pressure across the element.

However, these types of flowmeters are calibrated for a fixed composition and changes in the composition can dramatically affect the flow measurement. The flow rate (Q_v_) in a horizontal pipe can directly be derived by means of the Hagen-Poiseuille Eq. (1):

1 $${Q}_{v}=\frac{\Delta p\bullet \pi \bullet {R}^{4}}{8\bullet \mu \bullet L}$$

where Δp is the pressure difference of the inlet and the outlet, R the radius of the pipe (restriction), L the length of the pipe (restriction), µ the dynamic (absolute) viscosity of the gas.

For a certain LFE device, the parameters R and L are constant as they describe the geometry of the restriction. Consequently, the volumetric flow rate is a function of the pressure difference and the viscosity of the gas to be measured.

The viscosity describes the internal friction of a moving fluid. A fluid with low viscosity flows easily creating little friction when it is in motion. For gases at low pressures (0.1–10 bar) the viscosity is nearly independent of pressure but increases with increasing temperature.

Models for predicting the viscosity of pure components and mixtures have been described in detail in several articles, for example by Reid et al*.*^9^, Viswantha et al*.*^10^ and Monnery et al.^11^. As explained in^11^, viscosity models to calculate the viscosity of pure components can be divided into three main groups: theoretical, semi-theoretical and empirical models. The theoretical models are based on the kinetic theory of gases. One of the differences between the different theoretical models is if the interactions between molecules are taken into account or not. One of the most well-known models treating the interactions between colliding molecules is the Chapman-Enskog (CE) theory where the viscosity, ${\upeta }_{CE}$ is calculated according to Eq. (2).

2 $${\upeta }_{CE}=(5/16{\pi }^{1/2}) {\left[ \frac{({mkT)}^\frac{1}{2}}{{\sigma }^{2}{\Omega }^{\mathrm{2,2}}({T}^{*})}\right]}$$

where m is the mass of one molecule (g), k the Boltzmann’s constant (1.38048 × 10^–23^ J/K, T the temperature, ${T}^{*}$ the reduced temperature in K, $\sigma$ the collision diameter and ${\Omega }^{\mathrm{2,2}}({T}^{*})$ the collision integral.

Semi-theoretical models have a theoretical basis, but the parameters are adjustable and determined from experimental data. Corresponding states models do not necessarily require pure components values as input parameter. Empirical models are solely based on the correlation of experimental data.

In the same manner, an abundance of models has been proposed to calculate the viscosity of a gas mixture, ${\upeta }_{m}$. According to^11^, the equations for calculating the viscosity of gases mixtures can be categorized in 2 main groups:

- Mixing equations for pure component viscosities with a theoretical or an empirical basis. Examples with an empirical basis are the equations expressing the viscosity of a mixture as a sum of partial viscosities and where the interaction of dissimilar molecules is typically included in added terms. Simplified versions of the Chapman-Enskog theory to mixtures result in semi-theoretical equations.
- Equations where mixing rules are applied to parameters.

In this study, we have chosen five of these models with different levels of complexity. Three of the models chosen (Carr, Reichenberg and Wilke) belong to category (a) and the two other models (Chung and Lucas) belong to category (b).

Below, these models are summarily described, and some relevant equations are provided in order to underline which parameters are required for the calculation. A more complete description and additional equations can be found for example in the article from Monnery et al*.*^11^.

### Model of Carr^12^

The viscosity of the gas mixture, η_m_ according to Carr et al*.* is calculated from the momentum-weighted sum of the partial viscosities for each component i (Eq. 3).

3 $${\upeta }_{m}=\frac{\sum_{i}({y}_{i} . {\upeta }_{i} . \sqrt{{ M}_{i}})}{\sum_{i}({y}_{i} . \sqrt{{ M}_{i}})}$$

As it can be seen from Eq. (3), this model only requires for each component i of the mixture, the mole fraction ($${y}_{i})$$, the viscosity $$({\upeta }_{i})$$ and the molecular mass $${M}_{i}$$. This model is the simplest of the five models considered in this study.

### Model of Wilke^13^

In this model, the viscosity of the gas mixture, η_m_ is calculated according to Eq. (4):

4 $${\upeta }_{m}=\sum_{i=1}^{n}{\upeta }_{i} {\left[1+\frac{1}{{y}_{i}} \sum_{\begin{array}{c}j=1\\ j\ne i\end{array}}^{n}{\mathrm{y}}_{i}{\Phi }_{ij}\right]}^{-1}$$

The dimensionless constant ${\Phi }_{ij}$ is defined for each pair of components in a mixture according to Eq. (5):

5 $${\Phi }_{ij}=\frac{{\left[1+{\left(\frac{{\upeta }_{i}}{{\upeta
}_{j}}\right)}^{1/2}{\left(\frac{{\mathrm{M}}_{i}}{{\mathrm{M}}_{j}}\right)}^{1/4}\right]}^{2}}{{\left[8
{\left(1+\left(\frac{{\mathrm{M}}_{i}}{{\mathrm{M}}_{j}}\right)\right)}\right]}^{1/2}}$$

As for the Carr model, Wilke model only requires for each component i of the mixture, the mole fraction ($${y}_{i})$$, the viscosity $$({\upeta }_{i})$$ and the molecular mass $${M}_{i}$$.

### Model of Reichenberg^14,15^

Reichenberg (semi-theoretical model) proposed a simplified extension of the Chapman-Enskog theory to mixtures. The viscosity of a gas mixture, η_m_ is calculated according to Eq. (6).

6 $${\upeta }_{m}=\sum_{i=1}^{n}{\mathrm{K}}_{i} (1+2 \sum_{\begin{array}{c}j=1\\ \end{array}}^{i-1}{\mathrm{H}}_{ij}{\mathrm{K}}_{j} \sum_{j=1}^{n}\sum_{k=1}^{n}{\mathrm{H}}_{ij}{\mathrm{H}}_{ik}{\mathrm{K}}_{j}{\mathrm{K}}_{k})$$

To calculate $${\mathrm{K}}_{i}$$ (equation no given here but can be found in^11^) are required the mole fraction ($${y}_{i})$$, the viscosity $$({\upeta }_{i})$$ and the molecular mass $${M}_{i}$$.and the parameters $${\mathrm{H}}_{ij}$$.

The calculation of ${\mathrm{H}}_{ij}$ is usually done in several steps and the equations involved (which no given here but they can be found in^11^) require for each component i, the viscosity ${\upeta }_{i}$, the molecular mass ${M}_{i},$.the critical temperature ${\mathrm{T}}_{c,i}$, the critical pressure ${\mathrm{P}}_{c,i}$ and the dipole moment ${\upmu }_{i}$.

### Model of Chung^16^

The gas viscosity model of Chung is a combination of the Chapman-Enskog kinetic theory of viscosity and the empirical expression of Neufeld et al.^17^ for the reduced collision integral. The Chung model for gas mixtures is expressed as follows (Eq. 7).

7 $${\upeta }_{m}=26.693 .
{10}^{-4}\left[\frac{{\left({M}_{m}T\right)}^\frac{1}{2}}{{{\sigma
}_{m}^{2} {\Omega }^{\mathrm{2,2}}\left({T}_{m}^{*}\right)}}\right]
{f}_{m}({\omega }_{m}, {\mu }_{r,m},{K}_{m})$$

where ${M}_{m}$ is the molecular mass of the mixture, ${T}_{m}^{*}$ is the reduced temperature of the mixture, ${\Omega }^{\mathrm{2,2}}\left({T}_{m}^{*}\right)$ is the collision integral, ${\sigma }_{m}$ is the collision diameter of the mixture and ${f}_{m}$ is the corresponding states temperature reduction factor.

The model does not need pure component viscosities as input parameters and depends only upon the mixing rules for the pure component critical properties, the acentric factor ($\upomega )$, the Lennard–Jones potential parameters (ε/k and $\upsigma )$ and the dipole moment ($\upmu )$ which are the input data. The model requires performing cumbersome calculations which are not presented here but the calculations have been explained in detail in ^11^.

### Model of Lucas^18,19^

As for Chung model, this model does not need pure component viscosities and depend only upon the mixing rules for pure component critical properties. This model can be used to calculate the viscosity of pure gases. The parameters required are then the molecular mass (M), the critical pressure ($${P}_{C})$$, the critical temperature ($${T}_{C})$$,, the critical coefficient of compressibility ($${Z}_{C})$$. The model also requires the actual conditions of pressure and temperature. The viscosity of a gas mixture, η_m_ is calculated from the equation for pure gases according to Eq. (8). To estimate the viscosity of a gas mixture, instead of the pure components constants (M, $${P}_{C}$$, $${T}_{C}$$, $${Z}_{C}),$$ mixture constants ($${M}_{m}$$, $${P}_{Cm}$$, $${T}_{Cm}$$, $${Z}_{Cm })$$ are to be used. They are calculated from the composition of the mixture in accordance with the following mixing rules; Eqs. (9), (10), (11) and (12)

8 $${\eta }_{m}={(\eta \xi )}_{m}^{r,0}{f}_{P,m}^{0}{f}_{Q,m}^{0}\frac{1}{{\xi }_{m}}$$

where ${\xi }_{m}$ is the inverse viscosity, (Pa·s)^-1^, ${f}_{P,m}^{0}$ the low-pressure polarity correction factor, [–] and ${f}_{Q,m}^{0}$ the low-pressure quantum correction factor.

9 $${M}_{m}= \sum_{i}{y}_{i} {M}_{i}$$

10 $${T}_{cm}= \sum_{i}{y}_{i} {T}_{c,i}$$

11 $${P}_{cm}= \sum_{i}{y}_{i} {P}_{c,i}$$

12 $${Z}_{cm}= \sum_{i}{y}_{i} {Z}_{c,i}$$

where M_i_ is the molar mass of component i, y_i_ the mole fraction of component i in a gas mixture, T_c,i_: the critical temperature of component i in a gas mixture in K and P_c,i_: the critical pressure of component i in a gas mixture in Pa.

## Materials and methods

### Preparation of gaseous mixtures

Gaseous mixtures representative for different compositions of biogas, carbon dioxide and biomethane streams were prepared in one-liter stainless steel cylinders equipped with valves on both sides. The mixtures have been chosen for the representability of both biogas samples (binary mixtures 3 to 6 for biogas produced through digestion and multi-components 1, 2 and 3 for biogas produced in landfills) and biomethane samples (mixtures 1 and 2). The other mixtures have been chosen to test the method for gas containing high amount of carbon dioxide. The cylinders were first filled with a given pressure of pure methane. Secondly, pure carbon dioxide was added so the total pressure was around 10 bar, the composition of the mixture was then derived from the partial pressures of methane and carbon dioxide in the cylinders.

To verify the composition, each gaseous mixture produced was analysed with a Gas Chromatograph coupled with a Thermal Conductivity Detector (450-GC, Varian) equipped with two columns, a Hayesep Q, 80–100 Mesh, 1.8 m × 1/8′′ × 2.0 mm and a molecular Sieve 5A, 60–80 Mesh, 1 m × 1/8′′ × 2.0 mm. The gas cylinders were connected to the instrument via a pump and a 100 µL sampling loop. When the sampling loop was filled with the gas to analyse, a six-port valve was automatically switched, and the content of the loop was introduced into the columns connected in series. After 1.2 min, the molecular sieve column was bypassed, and methane and carbon dioxide were detected by the TCD. After 2.2 min, oxygen and nitrogen were detected using the two columns in series. The oven temperature was set constant at 60 °C, the detector was set at 100 °C with a filament temperature of 150 °C. The compositions of the mixtures are given in Table 1. Pure methane (CH_4_) and pure carbon dioxide (CO_2_) were also used in the study. Finally, two mixtures of CH_4_/CO_2_/N_2_ (50/20/30 v/v and 55/30/15 v/v) from Air Liquide and two mixtures from NPL (National Physical Laboratory) containing up to 9 components were also tested (composition given in Table 2).

**Table 1:** Composition of the binary gaseous mixtures used in this study.

Components (vol%)*	CH_4_	CO_2_
1	96.1 ± 0.2	3.9 ± 0.1
2	90.4 ± 0.2	9.6 ± 0.1
3	80.0 ± 0.2	20 ± 0.1
4	72.5 ± 0.4	27.5 ± 0.2
5	61.0 ± 0.4	39.0 ± 0.2
6	47.1 ± 0.3	52.9 ± 0.3
7	39.3 ± 0.2	60.7 ± 0.3
8	35.7 ± 0.2	64.3 ± 0.4
9	23 ± 0.1	77.0 ± 0.2
10	19.2 ± 0.1	80.8 ± 0.2

*The absolute measurement uncertainties (95% confidence interval) are given after the measured value.

**Table 2:** Composition of the multi-components gaseous mixtures used in this study.

Components (vol%)*	CH_4_	CO_2_	N_2_	O_2_	C_2_H_6_	C_3_H_8_	H_2_	CO	H_2_S
1	55.0 ± 0.4	30 ± 0.2	15 ± 0.1						
2	50.0 ± 0.3	20 ± 0.1	30 ± 0.2	–	–	–	–	–	–
3	47.76 ± 0.16	20.04 ± 0.1	25.06 ± 0.08	0.1035 ± 0.0014	2.026 ± 0.009	0.9772 ± 0.042	1.997 ± 0.011	1.987 ± 0.012	0.0508 ± 0.0006
4	11.017 ± 0.038	81.89 ± 0.4	5.959 ± 0.02	–	0.3018 ± 0.021	2.080 ± 0.0011	0.3095 ± 0.0037	0.3043 ± 0.0031	0.01004 ± 0.00011

*The absolute measurement uncertainties (95% confidence interval) are given after the measured value.

### Verification of the reference flowmeter (primary calibrator)

An automated (soap film) bubble flowmeter (Gilian Gilibrator-2 NIOSH Primary Standard for Air Flow Calibrator, St. Petersburg, USA) is used as reference for the flow rate measurements. The volumetric flow rate is determined by dividing a given volume by a time interval. The elapsed time is the interval needed for a soap film bubble to travel between two infrared sensors. The bubble is carried by the gas flow from the bottom to the top of the tube. The volume between the two infrared sensors is set accurately to a primary volume standard. A special soap solution is used to generate a bubble film stretched across the diameter of the flow cell tube. This specially compounded low residue soap is designed to provide high film strength and compatibility with the materials and gases used. Depending on the flow rate ranges, different flow cells are available. The smallest cell with a flow rate range of 1 to 250 mL/min was used in this study.

The performance of the reference flowmeter on other gases than air was verified using mass controllers (0–100 NmL/min, MC Series, Alicat, Tucson, USA) calibrated for pure methane and for pure carbon dioxide. The data showed a repeatability of ± 0.5% rel. and an accuracy of ± 1% rel. in the flow rate range 50 to 100 ml/min. For the other tests, the LFE flowmeter (from Alicat) was always set on methane.

### Experimental set-up

**Figure 1:**
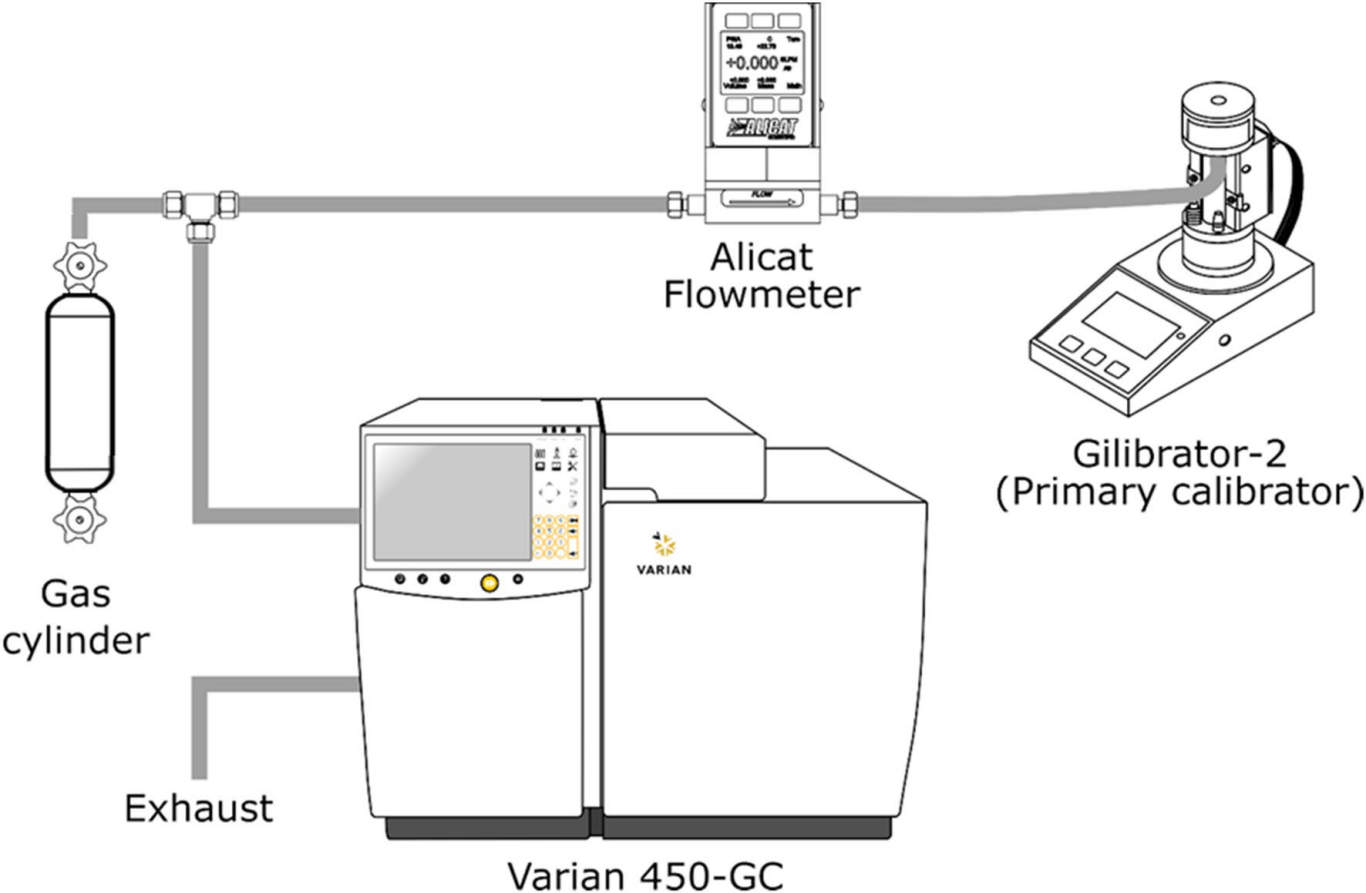
Scheme of the experimental set-up.

The two flowmeters were connected in series to the gas cylinders as shown in Fig. 1. A tee-connection was placed after the cylinders. Part of the flow from the cylinders went to the flowmeters and parallelly the gas from the cylinders was analysed with the GC/TCD using the analytical method described in the section “Preparation of gaseous mixtures”. The results of the gas composition measurements are given in Table 1 together with the measurement uncertainties for each component. The LFE flowmeter was pre-set on methane at different flow rate setpoints (between 25 and 100 NmL/min). The flow rate was measured in mL/min for the 14 different gas mixtures simultaneously with the reference flowmeter and the LFE flowmeter. The pressure through the set-up was measured by the LFE flowmeter (1002–1018 mbar). Each flow rate reported in the results is the mean of five consecutive readings within 5 min. The standard deviation observed for these readings was calculating to be less than 0.7% rel. at all flow rates tested. This low standard deviation is attributed to the fact that the LFE acts efficiently as an flow regulator and as long as the pressure of the gas is high enough, the flow rate is very stable.

### Calculations

For the three models that require as input data the absolute viscosity. at 25̊ C and 1013 mbar, the following values were taken from NIST (National Institute of Standards and Technology) database^20^: Methane, 110.76, carbon dioxide, 149.32, nitrogen, 178.05, oxygen, 205.50, ethane, 93.541, propane, 81.46, hydrogen 89.15, carbon monoxide, 176.49 and hydrogen sulphide, 123.87 μPoise. All the calculations were performed at a temperature of 25 °C and a pressure of 1013 mbar. As a comparison, the viscosities at 25 °C for pure methane and pure carbon dioxide were also calculated with the Chung and Lucas models. All the other input data were taken from NIST databases (REFPROP 10 data where available)^20^. The results are shown in Table 3.

**Table 3:** Calculated viscosity values for pure methane and pure carbon dioxide according to Chung, Lucas, compared to the viscosity from^20^.

Absolute viscosity (25 °C)	Chung	Lucas	^20^
CH_4_	111.46	110.06	110.76
CO_2_	147.74	150.91	149.32

The calculated viscosities obtained for the 14 gaseous mixtures with the five different models were then used to correct the reading on the LFE (Eq. (13) to account for the discrepancy due to the fact that the gas passing through the LFE was not pure methane but a mixture containing different volume fractions of up to nine components.

12 $$\mathrm{F}=\frac{100}{{\upeta }_{m}- {\upeta }_{CH4}}$$

where ${\upeta }_{m}$ is the calculated viscosity of the gas mixture and $\upeta$ the viscosity of pure methane.

## Results and discussions

### Viscosities of the gas mixtures with the different models

Using the five selected models, the viscosity of the 14 gas mixtures prepared in this study were calculated at 25 °C and 1013 mbar. The results are shown in Table 4.

**Table 4:** Viscosity of the gas mixtures obtained with the five different models.

Absolute viscosity (25 °C)	Carr	Wilke	Reichenberg	Chung	Lucas
Mixture 1	112.49	113.66	112.49	112.86	113.08
Mixture 2	114.97	117.57	114.98	114.99	116.83
Mixture 3	119.40	123.85	119.45	119.01	122.94
Mixture 4	122.50	127.77	122.60	121.96	126.84
Mixture 5	127.14	132.95	127.31	126.50	132.15
Mixture 6	132.53	138.09	132.81	131.86	137.67
Mixture 7	135.46	140.54	135.79	134.77	140.40
Mixture 8	137.14	141.58	137.13	136.09	141.59
Mixture 9	141.39	144.81	141.75	140.53	145.42
Mixture 10	142.74	145.67	143.07	141.79	146.47
Mixture 11	134.84	136.07	131.76	131.14	136.45
Mixture 12	139.22	138.93	136.06	135.32	140.54
Mixture 13	137.52	137.18	135.74	133.29	138.50
Mixture 14	147.36	148.23	147.39	146.17	149.67

**Figure 2:**
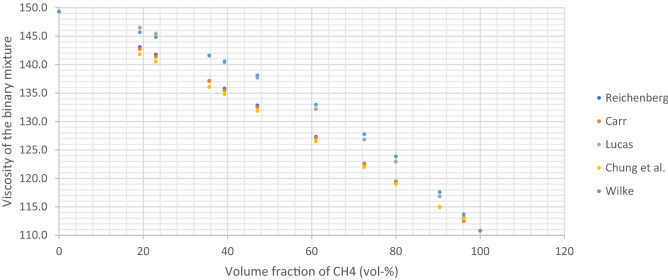
Viscosity of the binary gas mixtures from Table 1 calculated using five different models.

These results are also presented as Figures. Figure 2 shows the viscosity values obtained for the 10 binary CH_4_/CO_2_ mixtures (compositions in Table 1). The models gave rise to two distinct sets of viscosity values: Reichenberg, Carr and Chung on one hand (lower values) and Wilke and Lucas on the other hand (higher values). The highest relative standard deviations were found when comparing Wilke and Chung models for mixture 5 (3.5%), mixtures 4 and 6 (3.3%). All relative standard deviations are below 3%. The results indicated that all models showed a good agreement.

**Figure 3:**
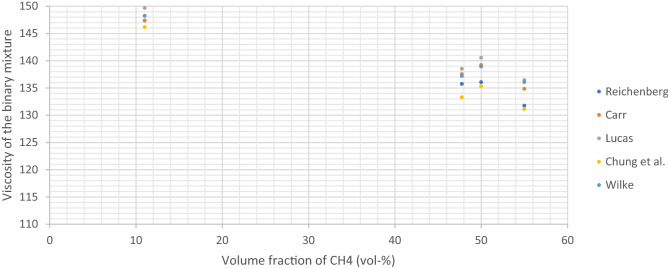
Viscosity of the multi-components gas mixtures from Table 2 calculated using five different models.

Figure 3 shows the viscosity values obtained for the four multi-component mixtures (compositions in Table 2). Chung consistently gave the lowest viscosity values and Lucas the highest when comparing the five different models. The relative standard deviations when comparing Lucas and Chung models for these four mixtures were 2.9, 2.8, 2.4 and 2.3% respectively, clearly indicating a good agreement between all the models.

### Comparison with the reference flowmeter

#### Binary mixtures

**Figure 4:**
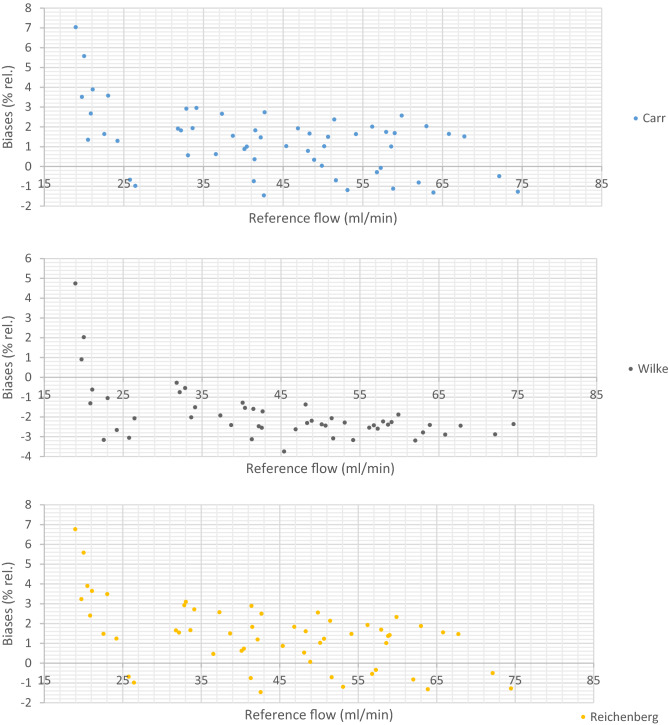

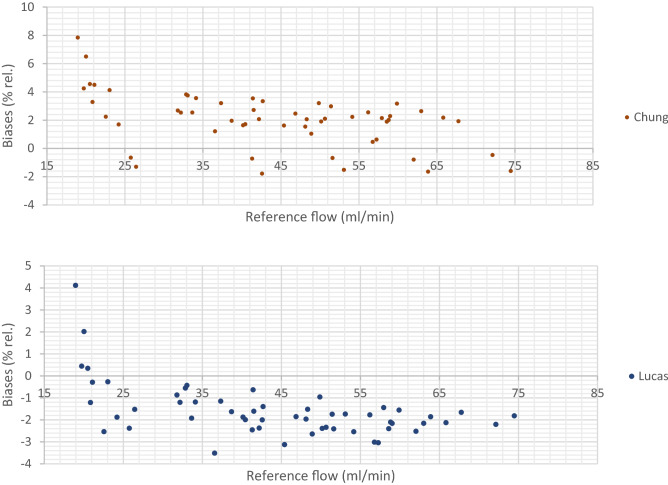
Biases between the flow rate measured with the LFE flowmeter corrected for the viscosity and the reference flow rate for the binary mixtures with the five different models: Carr, Wilke, Reichenberg, Chung and Lucas in this order.

The relative bias between the corrected LFE flow rate and the flow rate given by the reference flowmeter was calculated for all models at each setpoints. The data points were totally 50 for the binary mixtures; 10 values (for mixtures 1–10) at each of the five setpoints; 25, 40, 50, 60 and 70 NmL/min and 16 for the multi-component mixtures; four values for each at four setpoints (50, 70, 80 and 100 NmL/min). By comparing the biases, the models which best fit the values measured with the reference flowmeter can be determined. Positive biases indicate that the corrected flow rate measured by the LFE is overestimated compared to the flow rate given with the reference flowmeter and negative biases indicate that the flow rate measured by the LFE is underestimated compared to the flow rate measured with the reference flowmeter. The relative biases between the corrected flow rate measured with the LFE flowmeter and the reference flow rate are presented on Fig. 4 for the binary mixtures:

Large relative biases were observed for the setpoint 25 NmL/min with all models. However, the corresponding absolute biases are in the same order of magnitude than the absolute biases obtained at the higher flow rates. Due to the large absolute biases obtained at this flow rate, it is not recommended to work at this flow rate. For the discussion below, these 10 values have not been considered as this flow rate is actually lower than the flow rate range recommended for sampling biogas and biomethane on sorbent tubes^6,8^.

The Reichenberg and Carr models gave rise to the smallest biases (30 positive and 10 negative) with no biases above 3% rel. A maximal deviation of 2.92% rel. was obtained for mixture 8 at the setpoint 40 NmL/min with the Reichenberg model and a maximal bias of 2.95% rel. was obtained for mixture 6 at the setpoint 40 NmL/min with the Carr model. Lucas model gave rise to a maximal bias of − 3.51% rel. that was obtained for mixture 5 at the setpoint 40 NmL/min, and totally four values with a bias above 3% rel. were obtained All of the biases were negative. Chung model gave rise to a maximal bias of 3.81% (setpoint 40 NmL/min, mixture 8) and totally four values with a bias above 3% were observed. Most biases were positive (32 out of 40). Wilke model gave rise to a maximal bias of − 3.75% that was obtained for mixture 5 at the setpoint 50 NmL/min, and totally six values (at different setpoints) with a bias above ǀ3ǀ%rel. were obtained. All the biases were negative.

Globally, these results tend to indicate that Carr, Reichenberg and Chung models lead an overestimation of the flow rate except for the mixtures containing high amount fractions of methane (mixtures 1 and 2). On the opposite, Wilke and Lucas models tend to underestimate the flow rate. The results are also presented in Table 5.

**Table 5:** - Biases between the flow rate corrected for the viscosity with the five selected models and the flow rate measured with the reference flowmeter.

	Carr	Wilke	Reichenberg	Chung	Lucas
Positive biases	30	0	30	32	0
Negative biases	10	40	10	8	40
Number of Biases > ǀ3ǀ	0	6	0	4	4
n Biases between ǀ2ǀ and ǀ3ǀ	6	23	6	16	13
n Biases between ǀ1ǀ and ǀ2ǀ	20	8	20	14	21
n Biases between ǀ0ǀ and ǀ1ǀ	14	3	14	6	2

#### Multi-component mixtures

**Figure 5:**
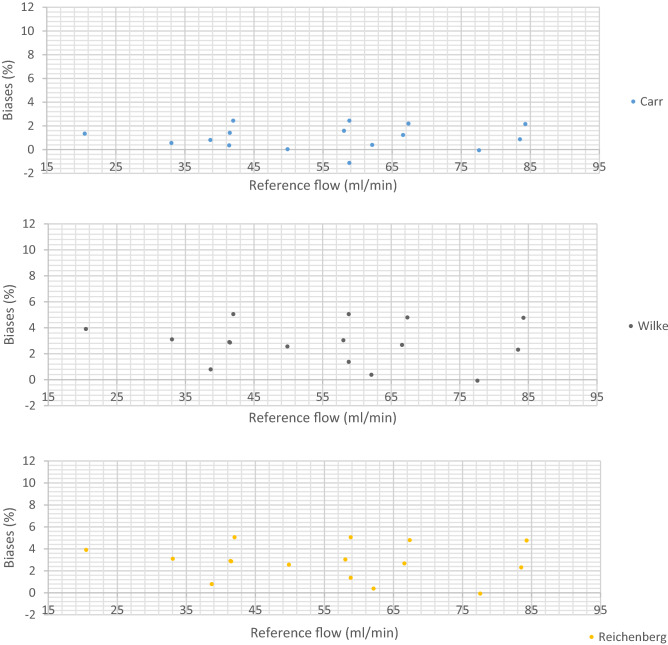

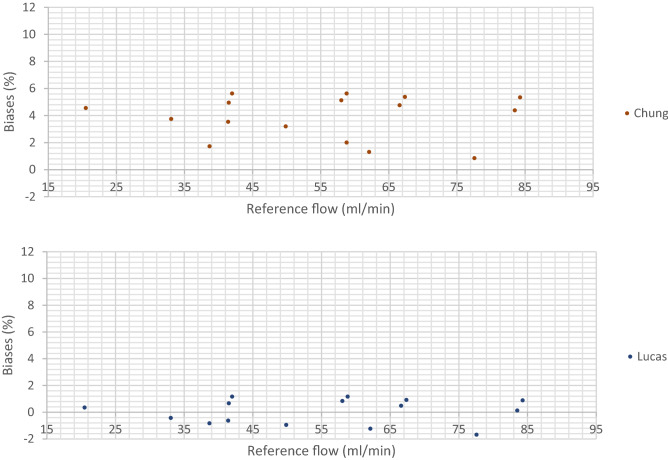
Biases between the flow rate measured with the LFE flowmeter corrected for the viscosity and the reference flow rate for the multi-component mixtures.

The relative biases between the corrected flow rate measured with the LFE flowmeter and the reference flow rate for the multi-component mixtures are presented on Fig. 5:

The results show that Lucas and Carr models clearly gave rise to the smallest biases. The Lucas model resulted in as many positive as negative biases with a maximal bias of − 2.10% which was obtained for mixture 11 at the setpoint 80 NmL/min. The Carr model resulted in 15 negative biases with a maximal bias of 2.44% which was obtained twice; for the mixture 12 at the setpoints 50 and 70 NmL/min. With the Reichenberg and the Wilke models, the maximal bias was 5.05% which was obtained twice; for the mixture 12 at the setpoints 50 and 70 NmL/min. All the biases were positive, which indicated that these models overestimated the flow rate. With the Chung model, the maximal bias was 5.63% also obtained for the mixture 12. Again, all the biases were positive, which indicated that the model also overestimated the flow rate.

## Conclusions

The study presents a method to correct flow rates measured with a LFE flowmeter pre-set on methane while used for gas mixtures of unknown composition at the time of the measurement. The method has several different possible applications inclusive for the sampling of biogas and biomethane onto sorbent tubes for conformity assessment. During the sampling of gases such as biomethane or biogas, the exact composition of the gas is often not known at the time of the sampling. The method proposed here implies to pre-set the LFE flowmeter on methane and to correct the value using a factor based on the later-determined viscosity of the gas once the composition has been precisely obtained.

In this study, five different models (Reichenberg, Wilke, Chung, Lucas and Carr) were evaluated to calculate the viscosity for binary mixtures of methane and carbon dioxide and for multi-component gases containing up to nine components commonly found in biogases. The method has been applied for flow rates ranging from 40 to 100 NmL/min.

The results of these evaluations showed that the factor using the viscosity of the mixtures calculated with the models of Reichenberg and Carr showed the smallest biases for binary mixtures. For multi-component mixtures, the best results were obtained when using the models of Lucas and Carr. It is worth noticing that the Carr model is the simpliest of all the models used in this study as it only requires for each component i of the mixture, the mole fraction ($${y}_{i})$$, the viscosity $$({\upeta }_{i})$$ and the molecular mass $${M}_{i}$$ and is calculated from the momentum-weighted sum of the partial viscosities for each component i.

Standard ISO16723-2 has yet no information or requirement regarding measurement uncertainties for the methods used to assess conformity assessment for all the parameters listed in the standard. The relative bias on the volume sampled with the method proposed here for the sampling of biogas and biomethane onto sorbent tubes.has to be taken into account and will impact the total measurement uncertainties of the method which includes the uncertainty due to the sampling as well as the uncertainty due to the analytical method.
